# A Year in the Life of the EU-CardioRNA COST Action: CA17129 Catalysing Transcriptomics Research in Cardiovascular Disease

**DOI:** 10.3390/ncrna6020017

**Published:** 2020-05-18

**Authors:** Emma Louise Robinson, Clarissa Pedrosa da Costa Gomes, Ines Potočnjak, Jan Hellemans, Fay Betsou, David de Gonzalo-Calvo, Monika Stoll, Mehmet Birhan Yilmaz, Bence Ágg, Dimitris Beis, Maria Carmo-Fonseca, Francisco J. Enguita, Soner Dogan, Bilge G. Tuna, Blanche Schroen, Wim Ammerlaan, Gabriela M. Kuster, Irina Carpusca, Thierry Pedrazzini, Costanza Emanueli, Fabio Martelli, Yvan Devaux, on behalf of the EU-CardioRNA COST Action

**Affiliations:** 1 Department of Cardiology, Cardiovascular Research Institute Maastricht (CARIM), Maastricht University, 6229 ER Maastricht, The Netherlands; b.schroen@maastrichtuniversity.nl; 2 Cardiovascular Research Unit, Luxembourg Institute of Health, L-1445 Strassen, Luxembourg; clarissa.gomes@uni.lu (C.P.d.C.G.); irina.carpusca@lih.lu (I.C.); 3 Institute for Clinical Medical Research and Education, University Hospital Centre Sisters of Charity, Zagreb 10 000, Croatia; ines.potocnjak@yahoo.com; 4 Biogazelle, 9052 Zwijnaarde, Belgium; Jan.hellemans@biogazelle.com; 5 Integrated BioBank of Luxembourg, L-3555 Dudelange, Luxembourg; fay.betsou@ibbl.lu (F.B.); wim.ammerlaan@ibbl.lu (W.A.); 6 Translational Research in Respiratory Medicine, University Hospital Arnau de Vilanova and Santa Maria, IRBLleida, 25198 Lleida, Spain; david.degonzalo@gmail.com; 7 Institute of Human Genetics, Genetic Epidemiology, University of Münster, 48149 Münster, Germany; mstoll@uni-muenster.de; 8 Department of Cardiology, Faculty of Medicine, Dokuz Eylül University, İzmir 35330, Turkey; prof.dr.mbyilmaz@gmail.com; 9 Department of Pharmacology and Pharmacotherapy, Semmelweis University, H-1085 Budapest, Hungary; agg.bence@med.semmelweis-univ.hu; 10 Pharmahungary Group, H-6722 Szeged, Hungary; 11 Centre for Clinical, Experimental Surgery, & Translational Research, Biomedical Research Foundation, Academy of Athens, 115 27 Athens, Greece; dbeis@bioacademy.gr; 12 Instituto de Medicina Molecular João Lobo Antunes, Faculdade de Medicina, Universidade de Lisboa, 1649-028 Lisbon, Portugal; carmo.fonseca@medicina.ulisboa.pt (M.C.-F.); fenguita@medicina.ulisboa.pt (F.J.E.); 13 Department of Medical Biology, School of Medicine, Yeditepe University, Istanbul 34755, Turkey; dogansoner@yahoo.com; 14 Department of Biophysics, School of Medicine, Yeditepe University, Istanbul 34755, Turkey; 15 Department of Biomedicine, University Hospital Basel and University of Basel, 4031 Basel, Switzerland; gabriela.kuster@usb.ch; 16 Department of Medicine, University of Lausanne Medical School, 1005 Lausanne, Switzerland; thierry.pedrazzini@chuv.ch; 17 National Heart and Lung Institute, Imperial College London, London SW3 6LY, UK; c.emanueli@imperial.ac.uk; 18 Molecular Cardiology Laboratory, Policlinico San Donato IRCCS, San Donato Milanese, 20097 Milan, Italy; fabio.martelli@grupposandonato.it

**Keywords:** COST Action, network, non-coding RNA, cardiovascular disease, translational research, interdisciplinary

## Abstract

The EU-CardioRNA Cooperation in Science and Technology (COST) Action is a European-wide consortium established in 2018 with 31 European country members and four associate member countries to build bridges between translational researchers from academia and industry who conduct research on non-coding RNAs, cardiovascular diseases and similar research areas. EU-CardioRNA comprises four core working groups (WG1–4). In the first year since its launch, EU-CardioRNA met biannually to exchange and discuss recent findings in related fields of scientific research, with scientific sessions broadly divided up according to WG. These meetings are also an opportunity to establish interdisciplinary discussion groups, brainstorm ideas and make plans to apply for joint research grants and conduct other scientific activities, including knowledge transfer. Following its launch in Brussels in 2018, three WG meetings have taken place. The first of these in Lisbon, Portugal, the second in Istanbul, Turkey, and the most recent in Maastricht, The Netherlands. Each meeting includes a scientific session from each WG. This meeting report briefly describes the highlights and key take-home messages from each WG session in this first successful year of the EU-CardioRNA COST Action.

## 1. Introduction

The EU-CardioRNA Cooperation in Science and Technology (COST) Action (CA17129; Catalysing transcriptomics research in cardiovascular disease) is a four-year international interdisciplinary network launched in 2018. COST (European Cooperation in Science and Technology) is funded by the European Union scientific frameworks, including the Horizon 2020. EU-CardioRNA is chaired by Yvan Devaux (Luxembourg Institute of Health, LU) along with Vice Chair Costanza Emanueli (National Heart and Lung Institute, Imperial College London, UK) and the conceptualization and formulation of the network was put together with Clarissa Pedrosa da Costa Gomes (now of Luxembourg Centre for Systems Biology, University of Luxembourg, LU). The Action kicked off in October 2018 with 37 participants from 21 countries. Today, it stands at 179 participants from 36 countries, including international partner countries such as Russian Federation, Singapore, Australia, Armenia and the United States of America.

By the time of the Maastricht meeting in February 2020, EU-CardioRNA had 168 participants, 46% of which are females and 28% are early careers investigators (ECIs, within 8 years of PhD or equivalent). More than half of leadership roles in the Action were held by female members.

Opportunities and activities within the EU-CardioRNA COST Action include short-term scientific missions (STSMs), which fund ECIs for a scientific exchange visit from a laboratory in one COST member state to that in another COST member state for up to three months. This is an opportunity to harness collaborations and facilitate exchange of techniques, infrastructure and samples between COST Action labs as well as enable mobility and enhance the career profile of ECIs.

Further small grants are given for ECIs based in inclusiveness target countries (ITCs) to attend international meetings. ITC conference grants give ECIs the opportunity to share their work and present to a wider audience, which may not be available to them otherwise. COST ITCs are Bosnia-Herzegovina, Bulgaria, Cyprus, Czech Republic, Estonia, Croatia, Hungary, Lithuania, Latvia, Luxembourg, Malta, Montenegro, Poland, Portugal, Romania, Slovenia, Slovakia, North Macedonia, Republic of Serbia and Turkey.

EU-CardioRNA participants met biannually in the first year of the COST Action, with scientific sessions during the meeting organised by each core working group (WG).

## 2. EU-CardioRNA Working Group Organisation

**Figure 1:**
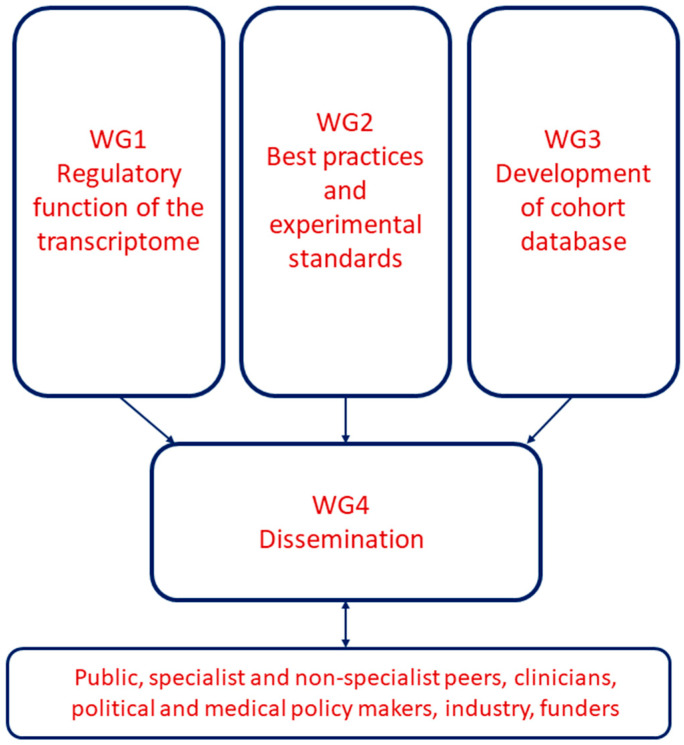
EU-CardioRNA Working Group organisation.

The EU-CardioRNA COST Action WG organisation is depicted in Figure 1. Working group 1 (WG1) “Regulatory function of the transcriptome” is led by Fabio Martelli (Policlinico San Donato, IT) along with Thierry Pedrazzini (co-Leader, Experimental Cardiology Unit, University of Lausanne, CH) and Emma Robinson (ECI co-Leader, Cardiology, Maastricht University, NL). WG1 aims to identify novel non-coding RNAs (ncRNA) and characterise their regulatory functions in cardiovascular pathophysiology, including their interactions with protein-coding transcripts. WG1 is the largest WG by number, with 109 members, and includes those members working in experimental fundamental and translational cardiovascular research.

The EU-CardioRNA Working Group 2 (WG2) “Best practices and experimental standards” focusses on the best practices for cardiovascular transcriptomic studies from sample collection and processing to the techniques used to study the transcriptome and the statistical analysis. Due to the low reproducibility and the large discrepancy of findings reported by independent groups, the tasks of WG2 are fundamental. The WG2 aims to review, propose and generate guidelines and protocols for sample and data processing, which ultimately can facilitate the comparison of the results from different laboratories. WG2 is led by Jan Hellemans (Biogazelle, BE), together with Fay Betsou (co-Leader, Integrated Biobank of Luxembourg (IBBL), LU) and ECI co-Leader David de Gonzalo-Calvo (Instituto de Investigaciones Biomedicas de Barcelona, SP).

Working Group 3 (WG3) “Development of cohort inventory” is led by Monika Stoll (University of Münster, DE), together with Mehmet Birhan Yilmaz (co-Leader, Dokuz Eylul University, TR) and ECI co-leader Bence Ágg (Semmelweis University, HU). The goal of WG3 is to create a user-friendly inventory of available cohorts and biobanks in the member states classified according to specific cardiovascular diseases (CVDs). After specifying the most important features of biobanks that should be collected in the inventory, a publicly searchable database with a web-based framework will be developed and populated with the appropriate details of the cohorts.

The EU-CardioRNA Working Group 4 (WG4), “Dissemination” is responsible for promoting and managing internal communication, external dissemination and knowledge exchange of the work of the COST CA17129 network members as well as opportunities provided by the network to support early careers in the field. WG4 currently has 16 members involved in promoting the activities of the action. Between October 2018 (the EU-CardioRNA kick off) and May 2019, WG4 was led by Clarissa Pedrosa da Costa Gomes (formerly of Luxembourg Institute of Health, LU). As of May 2019, the WG4 Leader and Science Communications Manager is Emma Robinson (Maastricht University, NL), who works together with co-Leader Gabriela Kuster (University hospital Basal, CH) and ECI co-leader, and the EU-CardioRNA scientific representative of Grant Holder Ines Potočnjak (University Hospital Centre Sisters of Charity, Zagreb, HR).

**Figure 2:**
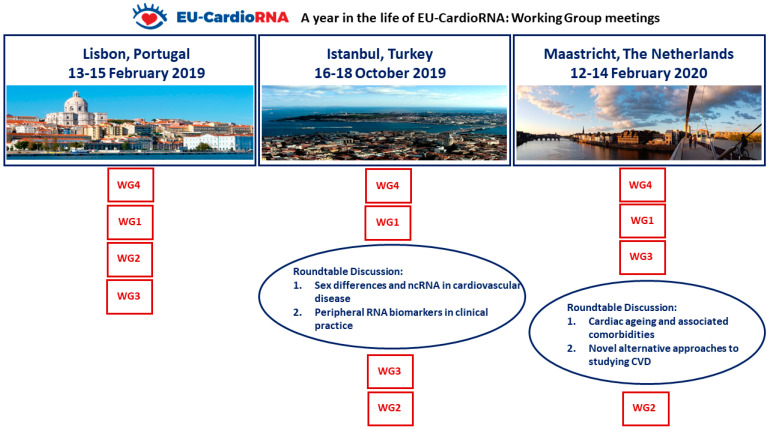
Organisation and evolution of the EU-CardioRNA COST Action working group meetings Feb 2019–Feb 2020. The order of sessions chronologically is demonstrated by the vertical order.

This comprehensive meeting report describes the content, discussions and highlights of scientific sessions for the three EU-CardioRNA COST Action WG meetings held between February 2019 and February 2020. An overview of the three meetings is shown in Figure 2.

## 3. Second EU-CardioRNA Working Group Meeting, Lisbon, Portugal

The second EU-CardioRNA WG meeting took place at the University of Lisbon, Instituto de Medicina Molecular (iMM) on 13–15 February 2019. The meeting was hosted and co-organised by Maria Carmo-Fonseca and Francisco Enguita, along with the Action Chair, Vice Chair and Clarissa Pedrosa da Costa Gomes.

## 4. WG1 Scientific Session

On the afternoon of Wednesday 13 February 2019, the CardioRNA COST Action WG1 scientific sessions commenced. Representing the relative size of WG1, which is comprised of more than two thirds of the Action’s members and participants, there were two WG1 scientific sessions, with five and six speakers, respectively. The WG1 co-leader, Thierry Pedrazzini, and ECI co-leader Emma Robinson chaired the sessions. The WG1 scientific programme included one ECI, 36% of presenters were female and 18% of speakers were from an ITC (Portugal).

The focus of WG1 is basic mechanisms and ‘Regulatory Function of the Transcriptome,’ with concentration on identifying novel ncRNAs and characterising their regulatory functions in cardiovascular pathophysiology. Under the umbrella of this common theme, members and participants in WG1 have a diverse range of expertise including bioinformatics, clinical research and biostatistics, biochemistry, molecular biology and experimental cardiology (pre-clinical models). In accordance with the goals of the EU-CardioRNA COST Action, the interdisciplinary nature of WG1 aims to accelerate translation of experimental data into clinical applications [1].

Topics discussed during the first session ranged from discussing the crossover in pathophysiological mechanisms between cancer and CVD (Carlo Gaetano, IT and Gabriela Kuster, CH) to the role of ncRNAs in risk factors and comorbidities of CVD, including atrial fibrillation, kidney disease and metabolic syndrome (Francisco Enguita, Dimitris Kardassis, GR, Timo Brandenburger, DE) [2,3].

The second part of the session exemplified the range of different tools and approaches being used to study transcriptomics in cardiovascular pathologies. This included talks on next-generation and single cell sequencing (scRNA-seq) (Emma Robinson, Andy Baker, UK), induced pluripotent stem cell-derived cardiomyocytes (Maria Carmo-Fonseca), and in vivo manipulation of ncRNAs using antisense technology (Johannes Winkler, AT) [4,5].

Other key and highly topical research areas discussed were the role of genetics in regulation of the cardiac transcriptome (Markus Scholz, DE) and sex differences in CVD and sex hormone regulation of ncRNAs implicated in cardiac pathophysiology (Susana Novella, SP) [6]. These hot topics in translational cardiovascular research will be explored in a focused manner in roundtable discussions at future meetings to initiate interdisciplinary approaches to better understanding and accelerate progress in these important and still ill-understood aspects of cardiovascular transcriptomics.

Importantly, new unpublished data, project and experimental plans for the future were shared with Action members and new ideas and collaborations sparked to raise the strength and impact of the proposed work.

This WG1 session summary demonstrates the heterogeneity and broad cross-section of expertise amongst WG members. We look forward to hearing more, as well as new data and project plans resulting from collaboration within and outside the Action, and lively scientific discussions on these topics in EU-CardioRNA meetings to come.

## 5. WG2 Scientific Sessions

The WG2 leader, Jan Hellemans, chaired the WG2 session on 14 February 2019. The presented topics demonstrated the heterogeneity of expertise among the WG2 members. Topics presented in the session ranged from microRNA (miRNA) quantification and applicability in cardiovascular pathology (Päivi Lakkisto, FI and David de Gonzalo-Calvo, SP) to good laboratory practices, reference materials and personal experience on RNA analysis (Christos Papaneophytou, CY, Fay Betsou, and Serdal Arslan, TR). Other topics presented were genetics and molecular mechanisms of disease (Leopoldo Laricchia Robo, IT).

The discussions of the different talks were focused on the experimental designs and the methodology used for RNA quantification. The audience proposed the presentation of unpublished and ongoing projects in future scientific sessions.

## 6. WG3 Scientific Sessions

The WG3 meeting was opened by ECI co-Leader Bence Ágg, who presented the most important advances in connection with WG3, covering the technical aspects of the planned cohort database, including the role of artificial intelligence (AI), as suggested by Mehmet Birhan Yilmaz.

Kanita Karaduzovic-Hadziabdic (BH) gave a general introduction about currently available AI technologies and a detailed summary of their work focusing on the utilisation of AI in the decision making for the diagnosis of CVDs. Bence Ágg presented two successful applications of a network theory-based, user-friendly software framework (miRNAtarget.com) for the comprehensive and unbiased analysis of miRNA transcriptomes [7,8]. With this software, they predicted and successfully validated multiple mediators central in the pathogenesis of hypercholesterolemia and systemic sensory neuropathy-induced myocardial dysfunction. Another possible application of AI was presented by Mitja Lustrek from Slovenia focusing on the application of classification trees in the prediction of outcome in heart failure (HF) patients based on the clinical parameters. Tim O’Brien (IE) summarised their work on the therapeutic application of mesenchymal stromal cells to reduce diabetic complications [9].

Important questions raised by WG Leaders Monika Stoll and Bence Ágg include which pathological entities should be included in the inventory, which clinical parameters (e.g., risk factors, echocardiography parameters, ventricular dimensions etc.) should be recorded, whose permission is required to include the cohort in the inventory and how transcriptomics data could be accessed by the potential collaborators in case of yet unpublished cohorts.

Planning of the tasks to be performed to implement the cohort inventory and achieve the deliverables of WG3 were also discussed by the members.

## 7. WG4 Session

Together with EU-CardioRNA’s Science Communication Manager, Emma Robinson and former WG4 Leader, Clarissa Pedrosa da osta Gomes, presented the communication strategy of this COST Action during the Lisbon meeting. The primary goals of EU-CardioRNA’s dissemination activities are to:

(1) Develop and support communication with the Action members.
(2) Inform external target groups about the challenges and promote the solutions proposed by this Action.
(3) Engage stakeholders and funding bodies.
(4) Encourage knowledge transfer.
(5) Facilitate the interaction with partners from the industry to maximise the impact of the Action’s outcomes.

EU-CardioRNA COST Action counts on the participation of researchers from different specialties, clinicians and representatives from the industry. Other target audiences to involve in the Action include European and National Cardiology and RNA Societies, scientists with a background in population science, systems biology and bioinformatics, specific standardisation bodies and policy makers. Disseminating the outcomes of the Action to the general public and selected lay interest groups is also a relevant aspect of the communication strategy. Since the network is multidisciplinary and has various stakeholders, dissemination will be adapted to each. The primary dissemination methods include an open-access dedicated website, biannual electronic newsletters, open activities organised by this Action to outside participants, presence at international scientific conferences, seminars and workshops, and publications in peer-reviewed journals.

## 8. Third EU-CardioRNA Working Group Meeting, Istanbul, Turkey

The third WG meeting was held at Yeditepe University in the beautiful city of Istanbul on 16–18 October 2019. The two-and-a-half-day meeting was hosted by Soner Dogan and Bilge Guvenc Tuna, both of the Yeditepe University School of Medicine,

For the first time, the biannual WG meeting was centred around two particular themes:

(1) Sex differences and ncRNA in CVD.
(2) Peripheral RNA biomarkers in clinical practice.

The goal of this inception is to try to create further focus, even within a relatively specialist field, to discuss and think about areas of current needs for advancements in understanding and standardisation or harmonisation of methodologies and approaches. Roundtable discussions were held with these themes, chaired and directed by experts working in the field. Keynote speakers were also invited with the focused topics in mind. In some cases, the roundtable discussion will be reviewed in separate articles.

## 9. WG1 Scientific Sessions

The WG1 scientific sessions in Istanbul were chaired by Fabio Martelli and Emma Robinson. One aspect addressed during WG1 sessions in Istanbul was the difference in CVDrisk, aetiology and gene expression according to sex. Jean-Francois Arnal (FR) kicked off the WG1 scientific session with an inspiring keynote talk summarising the state-of-the-art in the biology underlying sex differences in mammals. The three main ‘underlying causes’ of sex differences, those that are directly related to sex hormones and hormone receptors, the organisational or epigenetic memory of having been exposed to difference sex hormones in utero and the consequence of males and females having different chromosomal composition. Incomplete X chromosome silencing or X chromosome reactivation were discussed in depth. One of the X chromosomes is inactivated in early life through a crucial process called X-chromosome inactivation, mediated by the long non-coding RNA (lncRNA) Xist and polycomb repressor complex 2 (PRC2). Whilst incidence of CVD is lower in women than men, women are more predisposed to inflammatory disorders, such as autoimmune diseases (e.g., systemic lupus erythematosus (SLE) and multiple sclerosis), as well as metabolic and inflammation-associated diastolic dysfunction [10,11,12]. However, females also respond better to vaccinations [13]. There is statistical and mechanistic evidence to suggest that the effectivity and activity of the innate and adaptive immune system between the sexes are different. Some of this could be explained by activation of key X chromosome-encoded genes involved in the innate and adaptive immune response, including toll-like receptor 7 (TLR7) and Chromosome X Open Reading Frame 21 (CXorf21) [10,14]. The mechanism behind this biallelic X chromosome expression associated with immune disorders is unknown, but Xist and H3K27me3 localisation has been found to be altered in T-cells in females, in murine SLE models and in SLE patients [15]. It was also mentioned that sex needs to be considered in preclinical studies of CVD. Many historic and seminal studies in experimental cardiology have been performed in male animals. This is in part due to the conception that females give greater variability, so more animals needed to reach the same confidence limit, and oestrogens have a protective nature, making creation of models of CVD in females more difficult. The different stages of menstrual cycle in mice likely accounts for some of the perceived higher biological variability. We are moving towards a consensus that in all CVD preclinical and clinical studies, both males and females should be used, as results from studies in males may not apply to females. To overcome the confounding factor of a menstrual cycle in female mice, there are possibilities to assess the cycle stage or to sync cycles within a cohort of female mice [16]. The need to consider both males and females implicates that the number of subjects needed is more than doubled. These elements were discussed further in the roundtables.

Susana Novella (SP) reported on oestrogen-regulated miRNAs in endothelial cells that contain membrane-bound and nuclear oestrogen receptors, and the consequences it has on the transcriptome regulation in cardiovascular physiology, inflammation and angiogenesis. Particular attention was dedicated to miRNA-30b-5p and mitogen-activated protein kinases signalling [6]. In addition, the effect, expression and biology of androgens and androgen receptors, are just as important at studying the relative protection of women against pathological cardiac remodelling as investigating the role of oestrogens. Moreover, Joerg Heineke (DE) reported that androgens promote cardiac hypertrophy. Accordingly, androgen receptor knockout mice display lower heart hypertrophy upon angiotensin II (Ang II) treatment. The expression of all isoforms of the 5-α-reductase was induced in both human and mouse hypertrophic hearts. Accordingly, myocardial accumulation of its product, dihydrotestosterone (DHT), was increased too. Thus, the therapeutic potential in HF of the anti-androgen drug finasteride (a 5α-reductase inhibitor) that works by decreasing the production of DHT was explored [17]. In this study, in mice subject to transverse aortic constriction (TAC) treated with finasteride, cardiac hypertrophy, dilation, fibrosis and function were markedly improved, not only in male, but also in female mice, although to a lower extent. Similarly, in another study, finasteride treatment potently improved cardiac function after myocardial infarction (MI). Mechanistically, finasteride, by decreasing DHT, inhibited the pro-hypertrophic Akt-mTOR pathway. In addition, pathological cardiac gene expression was globally reversed by finasteride in mice after MI [18]. In patients with HF, a retrospective analysis showed that anti-androgenic therapy with finasteride for prostate disease was associated with attenuated cardiac hypertrophy [19]. Stephanie Bezzina Wettinger (MT) presented that in a clinical cohort Maltese Acute Myocardial Infarction (MAMI) study, the clinical studies can be standardised and common variables in human translational investigations can be controlled [20]. In blood collected from the MAMI study, phlebotomy tests are performed at the same time each day and the time since last meal, cigarettes and caffeine intake homogenised in human subjects to reduce confounding factors hampering the search for novel biomarkers for CVD [20].

In another study, Florence Pinet (FR) gave an exciting update on the European Research Area Network (ERA-Net) LIPCAR-HF project, investigating the effectivity of the lncRNA LIPCAR as a biomarker in left ventricular remodelling in chronic HF patients [21,22]. Mechanistically, LIPCAR localisation in extracellular vesicles was investigated. On the other hand, Blanche Schroen (NL) investigated the role of immune activation in heart failure with preserved ejection fraction (HFpEF). Schroen employed a mouse model of HFpEF (high-fat diet +Ang II) to investigate the role of the miRNA, miRNA-155. This miRNA is expressed in cardiac macrophages and displayed a detrimental role in heart inflammation and metabolic remodelling [23]. Mechanistically, miRNA-155 may stimulate Interferon-gamma Stat 1 signalling. Another speaker, Umut Inci Onat (TR), also discussed metabolic and inflammatory disease. Onat discussed the role of eukaryotic initiation factor 2 alpha (eIF2α) and the integrated stress response (ISR) in mouse and human atherosclerosis [24]. Endoplasmic reticulum regulates mitochondrial clearance by activating the eIF2α pathway, increasing oxidative stress that, in turn, prompts inflammasome activation and interleukin-1β secretion by diet lipids. Modulation of ISR to reduce organelle stress can prevent inflammasome activation by diet fats, indicating a strategy to reduce lipid-induced inflammation and atherosclerosis.

Rapid-fire early careers investigator talks for WG1 were lively and diverse. Novel sophisticated methods to study dynamics of disease have been developed based on existing next-generation sequencing data. In this session, Claudio Angione (UK) discussed machine learning for mechanistic insights into cell behaviour in cardio-metabolic syndrome, while Panagiotis Chouvardas (CH) presented a bioinformatics approach to identifying lncRNAs controlling cardiac fibroblast by overlaying numerous transcriptomics and epigenomics datasets. In addition, Julia Mester-Tonczar (AT) reported on preliminary but promising results on circular RNAs in a large animal model of ischemic HF. Furthermore, adding on the topic of sex differences in CVD, Robin Verjans (NL) presented the phenotypic characterisation of a murine model of cardiometabolic syndrome [25,26]. In this context, Ana Paes (ES) talked about the effect of estradiol on endothelial expression of miRNAs dysregulated in acute coronary syndrome patients, while Emma Robinson (NL) reported on Titin-antisense-1, a lncRNA acting as a pathophysiological mediator and sex-specific biomarker in HFpEF.

In summary, WG1 continues to grow, with the specialisation of members being highly multidisciplinary. WG1 members look forward to combining approaches towards holistic and robust translational cardiovascular research with greater implication and applicability to human health.

## 10. WG2 Scientific Session

The WG2 co-leader ECI, David de Gonzalo-Calvo, chaired the WG2 session in Istanbul. During the session, seven WG2 members presented their topics of interest. The topics included demonstrated the heterogeneity of backgrounds among the WG2 members: biobanking, industry and academia. The session was opened by Win Ammerlaan (LU), who presented the utility of quantitative PCR and droplet digital PCR in RNA biomarker discovery and validation. Then, Matthias Hackl (AT) discussed the preanalytical and analytical challenges in miRNA biomarker development. The best practices for analysing miRNAs in cell-free biofluids were proposed. In this context, Christos Papaneophytou (CY) summarised the challenges in using circulating miRNAs as biomarkers in CVD [27]. In addition, Rossienne Farrugia (MT) also described how to identify and control the preanalytical variables in a research setting. The advantages, disadvantages and potential pitfalls of RNAscope multiplex fluorescent in in situ hybridisation was discussed by Zoltan Varga (HU) [28]. Lastly, two ECIs presented their work in rapid fire sessions. First, Maarten Vanhaverbeke (BE) discussed the potential pitfalls in whole blood RNA profiling in acute MI [29]. Finally, David de Gonzalo-Calvo summarised the findings of an ongoing project which aims to analyse lipoproteins as carriers of miRNAs. On the other hand, the discussion was focused on the main topics of the WG2: best practices and experimental standards during the discussion session.

## 11. WG3 Scientific Session

Since the previous meeting in Lisbon, necessary steps to construct the cohort inventory database were defined as follows:

(1) Design the database structure and determine pieces of information needed from each cohort (Responsible person: Bence Ágg—Semmelweis University, Budapest, HU).
(2) Implement a user interface for the database with the variables decided in step 1 (Responsible people: Kanita Karaduzovic-Hadziabdic and Osman Gursoy—International University of Sarajevo, BH).
(3) Populate the database with actual cohort data (Responsible person: still to be selected)

During the summer of 2019, step 1 was completed, as the draft of the cohort inventory database structure was approved by WG3 after including some minor changes suggested by WG3 members. Concerning step 2, a significant progress has been also made to implement the database structure and a web-based user interface for it.

Presentations related to WG3 at the meeting in Istanbul was opened by ECI co-leader Bence Ágg and Mehmet B. Yilmaz. First, Bence Ágg presented the approved database structure for the cohort inventory database along with a detailed entity relationship diagram. The database design, besides basic characteristics of cohorts, details of available samples and omics datasets, includes the necessary data structures to specify pharmaceutical treatments, disorders, disease severity groups and comorbidities investigated in the cohorts. Then, Kanita Karaduzovic-Hadziabdic presented the initial stage of the database implementation (currently work in progress), which is currently available online on the International University of Sarajevo server. The web application is being developed by Osman Gürsoy, Web and Application Developer from International University of Sarajevo, BH. The website is intended to have different levels of access permissions, where the first two roles require a user login:

(1) IT Administrator/developer (has all privileges).
(2) (a) Database administrator (responsible for editing the following database information: list of professions, academic titles, gender types, study types, sample types, list of disease severity classification systems, list of disease severity stages, list of omics methods, and omics repositories).
  (b) Cohort leader administrator (and/or other registered cohort users), responsible for editing information related to cohorts such as: list of institutes, researchers and coordinators involved in the cohort, cohort centres, cohort drugs, sample diseases, etc.
(3) Everyone else, i.e., public.

During the discussion at the Istanbul meeting, it was noted that due to the COST regulations, the possibility of a default manager (administrators) may not be feasible, though, one centre provided a research-based potential solution. It was also decided to add a link to the database website in the COST Action’s official website. Finally, Mariann Gyöngyösi (AT) presented her data regarding the predictive role of noncoding RNAs in experimental ischemic HF for a collaborative project, that will likely be recorded in the cohort inventory database.

## 12. WG4 Session

The WG4 Leader, and Science communication manager, Emma Robinson, and ECI co-leader, and Scientific representative of the Grant Holder Ines Potočnjak, chaired the WG4 session in Istanbul. The communication strategy has progressed significantly since the previous meeting in Lisbon in February 2019. Emma Robinson kick-started the session with updates on Science Communication and Dissemination. Primarily, our Memorandum of Understanding (MoU)-based EU-CardioRNA project report, ‘Catalysing Transcriptomics Research in Cardiovascular Disease: The CardioRNA COST Action CA17129,’ was published on 29 March 2019 [1]. From the NcRNA journal (MDPI) webpage, the article had been viewed 1695 times and downloaded 999 times (metrics as of 16 October 2019). The EU-CardioRNA official logo was launched in April 2019 and our website went live in May 2019 (produced by Done Digital) [1]. Furthermore, our LinkedIn group has 31 members and has received regular updates including advertising official EU-CardioRNA WG and Management Committee (MC) meetings, performed STSMs, relevant scientific meetings, EU-CardioRNA special journal issues and joining of new members, such as that of International Partner Country (IPC) Singapore through MC Observer Roger Foo (Genome Institute Singapore and National University of Singapore). The Twitter page (@cost17129) has 22 followers and ResearchGate project page (CardioRNA EU COST Action) has 283 reads and 11 followers. Followers are approximately 50:50 from both within and outside the EU-CardioRNA COST Action.

Scientific publications published on behalf of the EU-CardioRNA COST Action included two or more member authors from at least two different COST member states or partner countries. Three scientific papers, in addition to the previously mentioned EU-CardioRNA project report, had been published (until 16 October 2019) [30,31,32].

Numerous dissemination independent talks, workshops, lectures and meeting sessions with Chairs and Speakers from the EU-CardioRNA COST Action were communicated in the session (complete list on the website). Topical Collection and Special Issues currently open for submissions that will be published on behalf of the EU-CardioRNA COST Action were advertised, including those in *NcRNA* “Regulatory RNAs in Cardiovascular Development and Disease” (Guest Editor Yvan Devaux) and in the *International Journal of Molecular Sciences* “RNAs in Cardiovascular Diseases-CardioRNA EU COST Action“ (Guest Editors: Carlo Gaetano, Fabio Martelli, Yvan Devaux) and “RNAs in Brain and Heart Diseases” (Editors: Yvan Devaux, Wolfram Doehner, Fabio Martelli, Inga Zerr). The final speaker for the WG4 session was an invited 30 min talk from Daniel Closa (Institute for Biomedical Research Barcelona and Spanish National Research Council) entitled ‘Dissemination for all.’ Daniel wonderfully, and in an engaging manner, highlighted how unscientifically founded news is communicated in the media and that we should take note of how these messages reach popular press and the eye and mind of the general public. He used recent topical examples from popular press and media sites demonstrating how fantastical news, such as natural wonder substitutes for conventional medicine, in particular when supported and communicated by a celebrity with role-model status, is attention grabbing and seductive. He quotes ‘Speak now or forever hold your peace.’ If scientists, doctors and clinical investigators do not take responsibility and step up to educate the public on the latest relevant research and scientifically validated health guidelines, then we risk attractive but false news prevailing, putting public health at risk. In the post-dotcom era of fast, available information that can spread far and wide through social media, this is a high threat to our quest to reduce morbidity and mortality rates from all forms of disease. Hurdles to overcome are training scientists to speak with journalists and non-specialist media in a way that will effectively gain interest and reach the public eye. Daniel left us with the challenge to create a memorable, catchy, yet scientifically-sound newspaper headline-like summary of our work that would potentially grab the attention and understanding of the general public.

## 13. Fourth Working Group and Management Committee, Maastricht, The Netherlands

The fourth WG meeting of the EU-CardioRNA COST Action took place on 12–14 February 2020, hosted by Emma Robinson and Blanche Schroen at the Maastricht University Medical Centre (MUMC+).

The two themes of the Maastricht meeting were:

(1) Cardiac ageing and associated comorbidities
(2) Novel alternative approaches to studying CVD.

**Figure 3:**
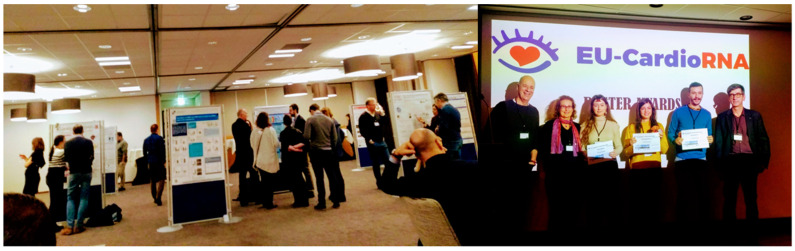
Photographs from the scientific poster session in Maastricht, 12 February 2020. Left: Lively discussion around the posters. Right: Poster prize winners with chairs of the three jury teams. Left-to-right: Thierry Pedrazzini (CH), Costanza Emanueli (UK), Teodora Barbalata (RO), Fabiana Martino (CZ), Francesco Ruberto (CH), Yvan Devaux (LU).

Roundtable discussions were also held that focused on these topics. Furthermore, for the first time, a call was put out for abstracts, from which up to 25 were selected for presentation during a lively and interactive scientific poster session on the first evening (Figure 3). The call for poster abstracts was targeted at ECIs. Three jury teams circulated and assessed poster presentations, with two runner-up prizes and one first prize, awarded to, Francesco Ruberto (CH), Teodora Barbalata (RO) and Fabiana Martino (CZ), respectively (Figure 3, right).

## 14. WG1 Scientific Session

The fourth COST Action meeting in Maastricht was the occasion for both established and junior participants to present ongoing projects. They represent only a small fraction of the work performed by the different participating laboratories but exemplify the quality of the research developed in the programme.

The WG session kicked off with a keynote lecture from Leon de Windt, Professor of Molecular Cardiovascular Biology and Chair of the Department of Molecular Genetics at Maastricht University. Leon gave a fascinating overview of the role of calcineurin and members of the family of Nuclear Factor of Activated T Cells (NFAT) transcription factors in initiation of cardiac hypertrophy. In particular, the different NFAT-mediated molecular pathways are necessary for initiation of cardiac hypertrophy in response to pathological stimuli compared with physiological stimuli. NFATc2 and NFATc3 play a major role in pathological cardiac remodelling and HF, whereas, whilst the Akt pathway is activated in response to physiological stimuli such as exercise, calcineurin-NFAT seems not to be necessary for physiological growth [33], thus defining some independent mechanisms in response to different stress signals in the heart.

Finally, Leon discussed impressive latest large animal data, showing vast improvements to cardiac function and attenuated ischemic remodelling when administered with anti-RNA therapy, promoting RNA therapies as a realistic treatment strategy for CVD [34].

RNA modifications in CVDs were first discussed by Konstantinos Stellos (UK). RNA contains over 140 distinct chemical modifications identified in both coding and noncoding RNAs. Of these, N6-methyladenosine (m6A) is the most prevalent. Additionally, N6-methylcytosine (m6C) should also be considered. Another important class of modifications is RNA editing, the most prominent of which is adenosine to inosine (A-to-I) deamination, catalysed in mammals by adenosine deaminase RNA specific 1/2 (ADAR-1/ADAR-2). Consistently, ADAR1 silencing strongly reduces A-to-I RNA editing. In human endothelial cells, editing events are primarily detected in Alu regions, in keeping with their ability to form double-stranded structures, a prerequisite for RNA editing. Among the most abundantly edited transcripts is Cathepsin-S, that bares two Alu sequences in its 3′-untranslated regions. RNA editing allows the recruitment of HuR RNA-binding protein and the stabilisation of Cathepsin-S mRNA, yielding higher protein levels. Interestingly, the extent of ADAR1-mediated Cathepsin-S RNA editing is associated with changes in Cathepsin-S levels in patients with atherosclerosis [35]. Along the same vein, correlation of A-to-I RNA editing and ADAR1 levels with inflammation is also observed in rheumatoid arthritis patients [36].

Then, Anne Yaël Nossent (NL) described her work on the miRNA epitranscriptome. Similar to mRNAs, modifications can affect miRNA precursors. In vascular cells, A-to-I editing in the seed sequence of pri-miRNAs and mature miRNAs is increased upon hypoxia. ADAR-dependent editing can modify target selection of mature miRNAs and increase their pro-angiogenic potential under hypoxic conditions. Moreover, miRNAs are also subject isomiRNA-formation. 5′-ISO-miRNA production is of particular interest for target selection. For instance, ISO-miR-411 is abundantly expressed in human vascular cells and targets different mRNAs from annotated miRNA-411 [37]. The ratio between ISO-miRNA-411 and miRNA-411 is modulated by ischemia. Importantly, ISO-miR-411 negatively influenced vascular cell migration, unlike miR-411. These data demonstrate that the same miRNAs can both inhibit and induce angiogenesis, depending on oxygen levels.

The role of miRNAs in cardiac metabolic disease was addressed by Mora Murri Pierri (ES). Mitochondria play crucial roles in energy metabolism but also, among others, in calcium homeostasis and apoptosis. In this context, miRNAs are crucial regulators of mitochondrial dysfunction. Many miRNAs are differentially expressed in brown versus white adipose tissue. During browning, miRNA-337-3p is upregulated and targets Twist1, a negative regulator of brown fat metabolism [38]. These findings have implications in our understanding of adipose tissue differentiation.

Panagiotis Chouvardas (CH) described a novel bioinformatic approach, named Lnc2MORE, developed for a systematic analysis of lncRNA action. Interrogating publicly available datasets allows genome-wide evaluation of lncRNA expression and regulation of cognate protein coding genes. A challenging view of lncRNA modes of action emerges from this analysis, in which lncRNAs exert predominantly Trans-regulatory function, and therefore, minimal Cis-regulatory effects, on protein coding gene expression. These findings need to be confirmed via comparing different subclasses/biotypes of lncRNAs.

Christoph Dieterich (DE) uses innovative approaches to study RNA splicing and translation in mouse models of heart disease. By combining ribosome-tagging (alpha-myosin-heavy-chainRibo-tag) and ribosome profiling (Ribo-Seq), the translated transcriptome of cardiomyocytes is evaluated in mice following TAC [39]. This technique allows identification of transcripts that are regulated at the translational level, demonstrating that translational control allows rapid changes in gene expression upon TAC. Particular attention is dedicated in this study to the role of upstream open reading frames (uORFs) as regulators of mRNA translation. Of note, the relevance of uORFs has been validated in published mass-spectrometry datasets identifying the corresponding uORF-encoded peptides. The way these peptides regulate translation, however, remains to be fully established. Finally, mRNAs controlled at the translational level are found to be associated with specific biological processes, such as metabolism, translation and protein quality control.

Simona Greco (IT) spoke about Beta-Secretase 1-antisense (BACE1-AS)-mediated mechanisms, and in particular, those that are shared between HF and Alzheimer’s disease (AD). BACE1-AS is a transcript with antisense orientation compared to BACE1, the key rate-limiting enzyme for the production of β-amyloid (βA), which is involved in the pathogenesis of AD [40]. In HF patients, BACE1-AS and BACE1 levels are increased in the ischemic myocardium, leading to intracellular βA accumulation [41]. Forced expression of BACE1-AS or βA administration in cardiovascular cells activates a common gene programme, resulting in apoptotic cell death. Mechanistically, BACE1-AS is predicted to bind and stabilises BACE1, and also a variety of other transcripts that are deregulated in both HF and AD. The potential of BACE1-AS and BACE1 as peripheral blood biomarkers in HF was also explored.

Roger Foo (Genome Institute, SG) used single cell analyses of human embryonic stem cell-derived cardiomyocytes to identify key regulatory lncRNAs of cardiomyocyte differentiation and maturation. A clustering approach allowed for identifying a novel lncRNA that is highly expressed and enriched in ventricular cardiomyocytes. Functional studies using stem cell-derived cardiomyocytes revealed this lncRNA as a new positive regulator of maturation through gene expression regulation.

The presentation by Johannes Backs (University of Heidelberg, DE) revolved around the regulation of histone deacetylase 4 (HDAC4) and its role in lipid and glucose metabolism. The lipid droplet-associated protein abhydrolase domain containing 5 (ABHD5) has been identified as the serine-protease cleaving HDAC4, yielding an N-terminal polypeptide (HDAC4-NT) [42]. During the response of the heart to stress, the NT fragment negatively controls myocyte enhancer factor-2 together with HDAC4 following calcium/calmodulin-dependent protein kinase IIinduced phosphorylation, allowing the expression of the hypertrophic programme. This is accompanied by a shift towards glucose metabolism, thereby implicating ABHD5 in substrate usage adjustment in stressed cardiomyocytes. Accordingly, ABHD5 deficiency in mice leads to lipid-storage disease with HF. In this situation, HDAC4-NT overexpression in cardiomyocytes improves heart function but has no effects on lipid accumulation, questioning lipotoxicity as one of the mechanisms underpinning the development of HF.

Katarzyna Fiedorowicz (PL) and Maria Carmo-Fonseca (PT) are studying cardiomyocyte differentiation from human-induced pluripotent stem cells (iPSCs). Fiedorowicz investigates the contribution of paracrine factors and epigenetic memory in the capacity of myoblast-derived iPSCs to give rise to mature and functional cardiomyocytes. Carmo-Fonseca uses iPSCs isolated from patients suffering from hypertrophic cardiomyopathy, in particular those carrying mutations in their myosin binding protein C3 gene, to evaluate RNA splicing-based therapy [43].

Finally, the fourth EU-CardioRNA WG meeting was closed with a keynote lecture from Eva van Rooij, full Professor of Molecular Cardiology at Utrecht Medical Centre and a Group Leader at the Hubrecht Institute.

The van Rooij group are renowned for identifying underlying cardiac disease mechanisms using scRNA-seq. Through scRNA-seq on murine and human healthy and injured hearts, there were able to identify novel sub cell-types as well as disease-specific cell types. Using scRNAseq, his group identified a new key modulator of fibroblast activation towards myofibroblast transformation in the injured heart. Inhibiting Cytoskeleton-Associated Protein 4 (CKAP-4) in fibroblasts stimulated with transforming growth factor beta (TGF-β) exacerbated the increase in gene expression relating to fibroblast activation [44]. Van Rooij talked through the limitations, caveats and considerations with the use of this ever-evolving technology. In particular, she discusses optimisation and using proper controls for isolation of single large rod-shaped cardiomyocytes for sequencing and how we are all learning with increased experience as we become familiar with the latest techniques.

Demonstrating how single cell technologies can be used to gain insight into the underlying cellular processes in cardiac pathophysiology, the lab have identified the transcription factor, Zeb2, as protective to cardiomyocytes against maladaptive cardiac remodelling in MI [44].

## 15. WG2 Scientific Session

Mathias Hackl (AT) presented several miRNA In-Process Control, which can be applied in RNA sequencing to reduce the inter-laboratory variability in miRNA profiling. Wim Ammerlaan of the IBBL Biorefinery Production Group focused on improved experimental reproducibility, which can be achieved by reduced pre-analytical variability. In transcriptomic studies, we can define multiple pre-analytic steps, e.g., Sample donation, transport conditions, sample processing. In a multicentre ring trial, seven different RNA extraction methods and spike-in-controls were evaluated. Significant differences in spike-in-recovery were found between the different extraction methods. Principle component analysis on the RNA sequencing data compared and contrasted between different kits revealed three distinctive populations of extraction methods. To address this, we recommend thorough documentation of the applied method, application of cross-platform internal controls and participation in external quality assessment schemes [45]. Next, Monica Marchese, Biomarker Validation Scientist at IBBL, focused on RNA biomarkers’ validation. In the light of the new Regulation (EU) 2017/746 on in vitro diagnostic (IVDR), many RNA biomarkers will be deemed as Companion Diagnostic (CDx) and will fall under class C. This means that before receiving Conformité Européene (CE) marking, it will be mandatory to present a biomarker dossier showing evidence of:

(1) Scientific validity (publication and/or patent).
(2) Analytical validity together with the determination of the pre-analytical characteristic of the biospecimens used, and traceability of values assigned to calibrators and/or control materials.
(3) Clinical performance.

This presentation highlighted the importance of testing RNA biomarkers for their robustness and the fitness-for-purpose of the method used for their discovery.

Louise Dalgaard (DK) emphasised the importance in identifying proper reference controls among endogenously expressed RNA species, and how sensitive experimental data are towards differences in reference control selection. Furthermore, to improve power and comparability between different cohorts and investigating research groups, it will be important to work together to identify more valid reference normalisation panels. The importance of External Quality Assessment for RNA extraction and Quality Control methods was presented by Olga Kofanova (LU). Clinical biospecimens are the most commonly used sample-type for research purposes but are often of poor quality given their susceptibility to uncontrolled and unrecorded pre-analytical variables. Within institutions that carry out biospecimen research, there is sometimes in-house quality control assays to guide researchers in their sample preparation, selection and processing decisions. To ensure that biological samples are suitable for their intended downstream applications and that their processing is fit for purpose, IBBL develops and implements external quality assurance (EQA) or proficiency testing (PT) schemes. The EQAs assess the efficiency of RNA sample preparation methods in terms of the quality of the resulting samples, to be used for downstream diagnostic or research purposes. The IBBL PT programme offers the following RNA-related PT analytical and processing schemes:

(1) RNA Quantification and Purity
(2) RNA Integrity
(3) Cell Free RNA (cfRNA) Extraction from Plasma
(4) RNA Extraction from Whole Blood
(5) RNA Extraction from Formalin Fixed Paraffin EmbeddedMaterial
(6) Total RNA Extraction from Frozen Tissue

The PT programme allows internal and external laboratories to validate their routine processing and analytical methods, compare their performance to that of others, comply with normative requirements, gain credibility and visibility, improve their performance and prove their consistency. Finally, María Laura Garcia Bermejo (SP) emphasised the importance of biomarker validation and the participation in large European networks, such as the European infastructure for translational medicine (EATRIS) [46].

## 16. WG3 Scientific Session

Following the meeting in Istanbul in October 2019, where the initial database structure and initial implementation of the database was discussed and approved, Kanita Karaduzovic-Hadziabdic and Osman Gürsoy (BH) implemented the web application, which is now in its pre-final stage and ready for fine-tuning.

The WG3 presentations in Maastricht addressed the remaining questions concerning the features to be curated within the database, the curation of the database itself, database security according to COST regulations as well the database design and fine-tuning prior to completion. Therefore, following the introduction of the goals of the session by Yvan Devaux and Monika Stoll, short presentations by Bence Ágg and Kanita Karaduzovic-Hadziabdic on the above-mentioned questions, the majority of the session was dedicated to discussion among the COST Action participants in order to finalise the database in a timely fashion.

Bence Ágg particularly addressed the issues related to the curation and population of the database, which, with the help and commitment of vice-rector of Semmelweis University, Peter Ferdinandy, will be curated and supported by Semmelweis University, Budapast, Hungary. The same counts for the clarification of legal issues, such as data protection (GDPR) and access to the World Health Organization data. The entire COST Action group expressed their gratitude towards Semmelweis University for their commitment to making the database a success. In the following, Kanita Karaduzovic-Hadziabdic, demonstrated the prefinal version of the database including the web interface, and started the discussion on improvements for utility as well as content. It was agreed that the database is already in very good shape but requires some additional improvements and testing in order to improve the search features and to make the database as user-friendly as possible. In the coming weeks, the work will be done on the additional features of making the database more user-friendly. After this step, further suggestions of fine-tuning of the database will be performed by the individual members of the COST Action in order to provide feedback and to discover potential glitches and bugs prior to going public. Overall, the project is well on track to meet the deadline for completion.

## 17. WG4 Session

The WG4 session in Maastricht kicked off with an update from the Science Communication Manager and WG4 Leader, Emma Robinson. The EU-CardioRNA website was launched in May 2019 (https://cardiorna.eu/) and receives regular (>once weekly) updates on the news page, including open opportunities for positions and grant applications from within and outside the Action itself, new publications or projects from our members and details of relevant upcoming meetings. EU-CardioRNA and related meetings, workshops and training schools are displayed on the Events calendar page. The website also hosts our participant database, a closed portal for members to create a portfolio with their contact information, upload their curricula vitae, expertise and publications. One hundred and eleven members have created a personal profile (as of 14 February 2020), enabling EU-CardioRNA COST Action participants to search for colleagues within the Action with certain expertise for collaboration and grant application partnerships. New to the website as of January 2020 is a Collaborations tab, displaying links to associated societies and networks with whom EU-CardioRNA has formed official partnerships. This includes the prestigious European Society of Cardiology Working Group on Stroke, formed on the joining of Wolfram Doehner to the EU-CardioRNA COST Action, the CardioLINC network and the EU-Cardioprotection COST Action, chaired by our MC member for Hungary, Peter Ferdinandy.

**Figure 4:**
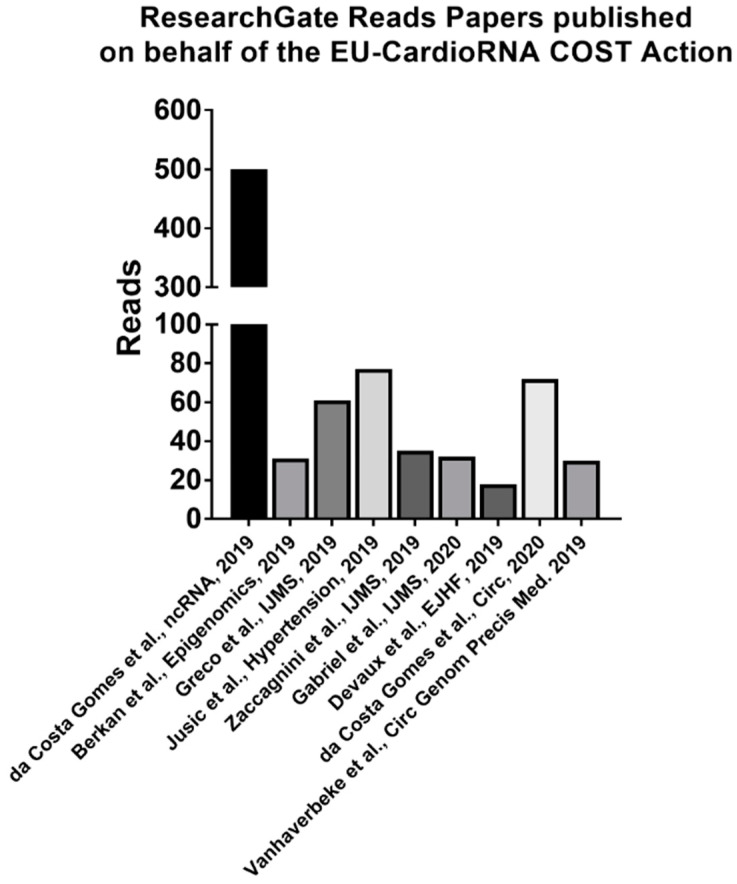
Running count of the number of reads for each paper published on behalf of the EU-CardioRNA COST Action from the ResearchGate Project as of 11 February 2020. https://www.researchgate.net/project/CardioRNA-EU-COST-Action.

The social media pages are also growing in followers, now with 43 LinkedIn group followers, 39 Twitter followers and 17 followers, 57 collaborators on the ResearchGate project (CardioRNA EU COST Action). The ResearchGate CardioRNA EU COST Action project has had 375 reads and manuscripts published on behalf of the EU-CardioRNA COST Action (two or more EU-CardioRNA participant authors from at least two or more COST member states) have 500 or more reads in total (Figure 4).

One of our most notable dissemination topics was the publication of the first EU-CardioRNA COST Action state-of-the-art in-depth review article in Circulation on 28 January 2020, which received more than 3300 views and 47 likes on LinkedIn (14 February 2020).

Looking ahead, the creation of a short, simple project-explainer animated video was discussed amongst the MC and budgeted into the next grant period spending for dissemination (GP3). This can be shared through the COST Association YouTube channel as well as on our own website home page and social media profiles and should be accessible to non-specialists and non-scientists.

The final WG4 talk was from the Project Manager at the Luxembourg Institute of Health, Irina Carpusca, giving her expert insights into how to manage an EU project. Building consensus-oriented decision-making processes based on communication and openness with all partners is key. One has to be aware of culture differences in communication speed and strategy in managing an international project and tailor the style of communication accordingly, where possible. Frequent though targeted communication with all partners and with the Project Officers ultimately helps with building trust and increases management efficiency.

## 18. Conclusions

Throughout the first year of WG meetings of the EU-CardioRNA COST Action, the meetings have evolved and grown in size, with the first meeting in Lisbon playing host to 54 attendees, and the most recent meeting in Maastricht welcoming 78 participants from 33 different countries.

With the growing number of participants with diverse skills, knowledge and approaches, WG meetings have been given focus, with two themes per meeting, and with the aim of focusing on key unmet questions and needs in translational cardiovascular research. Through the scientific sessions and roundtable discussions, the Action brings together researchers from different disciplines and backgrounds to catalyse the progress in using transcriptomics and ncRNA research to further knowledge, understanding, diagnostic and prognostic tools and treatments for CVD We look forward to another year with new as well as familiar faces, of successful collaborations and scientific exchange within EU-CardioRNA.

## Relevant Twitter Handles

EU-CardioRNA COST Action @cost17129
Dimitris Beis @baybeis
Emma Robinson @Robinson7L
Ines Potocnjak @ines_potocnjak
Daniel Closa @nielo40

## Important Websites

https://cardiorna.eu/; https://www.researchgate.net/project/CardioRNA-EU-COST-Action.

## Abbreviations

AI	Artificial intelligence
COST	Cooperation in Science and Technology
CVD	Cardiovascular disease
ECI	Early careers investigator
HF	Heart failure
IBBL	Integrated BioBank of Luxembourg
ITC	Inclusiveness target country
HFpEF	heart failure with preserved ejection fraction
iPSCs	Induced pluripotent stem cells
lncRNA	Long non-coding RNA
MI	Myocardial infarction
miRNA	MicroRNA
ncRNA	Non-coding RNA
scRNA-seq	Single cell RNA sequencing
STSM	Short-term scientific mission
TAC	transverse aortic constriction
WG	Working group
